# Obituary

**Published:** 1904-03-15

**Authors:** 


### OBITUARY.

### JAMES R. STEWART.

James R. Stewart, a graduate of theDental Department of the University of Michigan, class of 1902, died of pneumonia, at Coldwater, Mich., January 31st, 1904. He was a clever student and his many friends will be grieved by his death.

### DR. WILLIAM D. SAUNDERS.

Dr. William D. Saunders, of Grand Rapids, committed suicide in Ann Arbor, the 27th of January, 1904, because of ill health. Dr. Saunders was a graduate of the Dental Department of the University of Michigan, class of 1887.

